# Supplementary material for: A Sol-Gel/Solvothermal Synthetic Approach to Titania Nanoparticles for Raman Thermometry
Source: Sensors (Basel). 2023 Feb 26;23(5):2596. doi: 10.3390/s23052596 (PMC10007076; doi:10.3390/s23052596)
Supplement: Supplementary file 1 [file sensors-23-02596-s001.zip › sensors-2224770-supplementary.pdf]

SUPPLEMENTARY MATERIALS

S1. Samples prepared with different types of syntheses

Table S1. List of all samples prepared with the syntheses.

ETHYLENE GLYCOL	ACID	BASIC
TP12	TP22.1	TP26.7
TP13	TP22.11	TP26.8
TP10	TP22.2	TP26.9
TP11	TP22.3	TP21.1
TP14	TP22.4	TP21.2
TP15	TP22.8	TP21.3
TP18	TP22.10	TP21.4
TP19	TP25.1	TP21.5
TP23.1	TP27.1	TP21.6
TP16	TP22.7	TP25C
TP16.2	TP27.2	TP25S
TP17	TP25.2	TP25T
TP23.2	TP22.9	TP25T1
TP23.3	TP22.5	TP25T2
TP23.4	TP25.3	TP20
TP6	TP25.4	
TP7	TP25.5	
TP7_n2	TP25.6	
TP7.2	TP25.7	
TP7.3	TP25.8	
TP8	TP25.9	
TP9		
TP23.5		
TP26.3		
TP7_L		
TP8_L		
TP10_L		
TP23.6		
TP26.4		
TP26.5		
TP26.6		

S1.1. Ethylene Glycol Synthesis Samples

Table S2. List of samples prepared with the ethylene glycol synthesis and the corresponding experimental conditions.

Sample	$\frac{mol\,(H_2O)}{mol\,(Ti(OiPr)_4)}$	$\frac{mol\,(TEAOH)}{mol\,(Ti(OiPr)_4)}$	T [°C]	Time [h]	Notes
**TP12**	min *	0.22	180	24	Nonseparable Gel
**TP13**	min *	0.22	150	24	Nonseparable Gel
**TP10**	7	0.22	180	24	Nonseparable Gel
**TP11**	7	0.22	150	24	Nonseparable Gel
**TP14**	7**	0.22	150	24	Nonseparable Gel
**TP15**	7***	0.22	150	24	Nonseparable Gel
**TP18**	7	0.42	180	24	Nonseparable Gel
**TP19**	7	0.105	180	24	Nonseparable Gel
**TP23.1**	68.5	4.5	150	24	Crystalline Powder
**TP16**	75	0.22	180	24	Crystalline Powder
**TP16.2**	75	0.22	180	12	Crystalline Powder
**TP17**	75	4.5	180	24	Crystalline Powder
**TP23.2**	75	4.5	150	24	Crystalline Powder
**TP23.3**	100	4.5	150	24	Crystalline Powder
**TP23.4**	125	4.5	150	24	Crystalline Powder
**TP6**	150	4.5	180	24	Crystalline Powder
**TP7**	150	4.5	180	24	Crystalline Powder
**TP7_n2**	150	4.5	180	24	Crystalline Powder
**TP7.2**	150	4.5	180	5	Crystalline Powder
**TP7.3**	150	4.5	180	12	Crystalline Powder
**TP8**	150	4.5	150	24	Crystalline Powder
**TP9**	150	4.5	150	24	Crystalline Powder
**TP23.5**	150	4.5	150	24	Crystalline Powder
**TP26.3**	150	4.5	150	24	Crystalline Powder
**TP7_L**	150	4.5	130	24	No precipitation
**TP8_L**	150	4.5	130	24	No precipitation
**TP10_L**	150	0.22	130	24	No precipitation
**TP23.6**	175	4.5	150	24	Crystalline Powder
**TP26.4**	346	4.5	150	24	Crystalline Powder
**TP26.5**	708	4.5	150	24	Crystalline Powder
**TP26.6**	1431	4.5	150	24	Crystalline Powder

* Minimum amount of H_2O contained in the 35% TEAOH solution; **Titanium (IV) butoxide; **diethylene glycole.

As reported in the notes in Table S2, in some cases, there have been problems that have affected the success of the syntheses.

1) At low treatment temperatures (130°C, TP7_L, TP8_L, TP10_L samples) it was not possible to obtain a precipitate that could be separated by centrifugation, indicated with "*no precipitation*"; attempts at precipitation in water or acetone have been ineffective in solving this problem.

2) The formation of a persistent gel to hydrothermal treatment, which occurs with small quantities of peptizer (r<4.5, TP10, TP11, TP18, and TP19 samples), made it impossible to obtain nanoparticles, indicated as 'non-separable gel'. To solve this drawback, the reaction product has been transferred to a beaker and heated on a plate at 150°C to allow the evaporation of ethylene glycol (flash point in closed containers: 111°C at 1,013 hPa).

With a subsequent heating in a muffle at 200°C, a solid, dirty with organic residue was obtained, which can be ground with mortar and pestle. However, this recovery procedure, in addition to complicating the synthesis process, did not allow one to obtain crystalline material.
3) TP6, TP7 and TP7_n2 are sample prepared with the same synthesis on different days, at 180°C. The use of this temperature, with respect to 150°C, do not increase the quality of the material.
4) TP8, TP9, TP23.5 and TP26.3 are sample prepared with the same synthesis on different days.

S1.2. Acid Synthesis Samples

For acid syntheses, the idrothermal treatment has been set at a temperature of 150°C for a time of 24 h. The reactions have been divided into two main groups. In the first, the value of the ratio between the moles of acid and the moles of TTIP is equal to 210, in the second, the ratio is brought to a value of 105, the halving of the amount of acetic acid allows the hydrolysis ratio to increase in a wider range, comparable with other synthesis (reported in Table S3).This choice also makes the consumption of acetic acid lower and consequently makes the synthesis more sustainable.

Table S3. List of samples prepared with acid synthesis and the corresponding experimental conditions.

Sample	$\frac{mol\ (H_2O)}{mol\ (Ti(O^iPr)_4)}$	$\frac{mol\ (CH_3COOH)}{mol\ (Ti(O^iPr)_4)}$
**TP22.1**	1.66	210
**TP22.11**	3.32	210
**TP22.2**	4.15	210
**TP22.3**	16.6	210
**TP22.4**	41.5	210
**TP22.8**	1.66	105
**TP22.10**	3.32	105
**TP25.1**	3.32	105
**TP27.1**	3.74	105
**TP22.7**	4.15	105
**TP27.2**	10.38	105
**TP25.2**	16.6	105
**TP22.9**	16.6	105
**TP22.5**	41.5	105
**TP25.3**	75	105
**TP25.4**	125	105
**TP25.5**	150	105
**TP25.6**	225	105
**TP25.7**	346	105
**TP25.8**	708	105
**TP25.9**	1431	105

S1.2. Basic Synthesis Samples

For the basic syntheses, the idrothermal treatment has been set at a temperature of 150°C for a time of 24 h. The reactions have been performed at a buffered pH 9.5 obtained dissolving an ammonium salt in an ammonia; the salt used to create the buffer solution was varied to check whether the anion of the ammonium salt had an effect on NP extraction, on the polymorph obtained and on morphology.

Table S4. List of samples prepared with basic synthesis and the corresponding experimental conditions.

Sample	$\frac{mol\ (H_2O)}{mol\ (Ti(O^iPr)_4)}$	$\frac{mol\ (NH_3)}{mol\ (Ti(O^iPr)_4)}$	$\frac{mol\ (NH_4Cl)}{mol\ (Ti(O^iPr)_4)}$	Buffer
**TP26.7**	16.6	6.8	3.9	NH_3/NH_4Cl
**TP26.8**	41.5	17.1	9.8	NH_3/NH_4Cl
**TP26.9**	75.0	30.9	17.8	NH_3/NH_4Cl
**TP21.1**	128.7	5.4	3.1	NH_3/NH_4Cl
**TP21.2**	164.9	6.9	4.0	NH_3/NH_4Cl
**TP21.3**	225.2	9.4	5.4	NH_3/NH_4Cl
**TP21.4**	345.9	14.4	8.3	NH_3/NH_4Cl
**TP21.5**	707.9	29.5	17.0	NH_3/NH_4Cl
**TP21.6**	1431.8	59.7	34.4	NH_3/NH_4Cl
**TP25C**	225.2	9.4	5.4	NH_3/NH_4Cl
**TP25S**	225.2	9.4	5.4	$NH_3/(NH_4)_2SO_4$
**TP25T**	225.2	-	-	TEAOH No Buffer
**TP25T**	225.2	-	-	TEAOH No Buffer
**TP25T1**	225.2	-	-	TEAOH No Buffer
**TP25T2**	150	-	-	TEAOH No Buffer
**TP20***	21728.4			NH_3 No Buffer

* Without buffer, performed at 180°C.

Samples TP25C and TP25S alows to verify the effect of the Cl^- or SO_4^{2-} anion on the final result. A further test (TP25T sample) was carried out in a basic non-buffered environment, using the TEAOH peptizer to basify and avoid the aggregation of the obtained nanoparticles. Since TP25T synthesis led to the formation of rutile, further syntheses were made (samples TP25T1 and TP25T2) by decreasing the hydrolysis ratio to verify which polymorph was produced.

Sample TP20 has been synthesized in an aqueous ammonia solution according to the hydrolysis ratio obtained from the reference [Cho, C. H.; Han, M. H.; Kim, D. H.; Kim, D. K. Morphology Evolution of Anatase TiO2 Nanocrystals under a Hydrothermal Condition (PH=9.5) and Their Ultra-High Photo-Catalytic Activity. Materials Chemistry and Physics 2005, 92 (1), 104–111. https://doi.org/10.1016/j.matchemphys.2004.12.036.] in the absence of ammonium salts and in an unbuffered environment. For this test, the temperature of 180°C has been chosen because it was closest to that of the article.

S2. Optical and morphological Characterization of Samples

S2.1. Ethylene glycol synthesis Samples

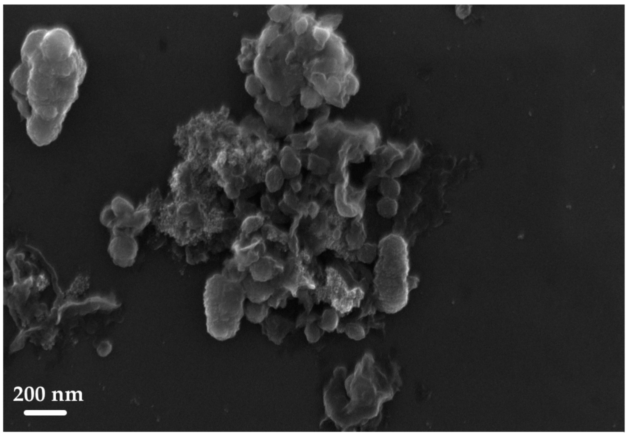

Figure S1. SEM image of TP23.3 sample. At *r*=100 the material is organized in micrometric aggregates, out of which nanoparticles of various morphology can be identified, with a size distribution that extends between 20 and 200 nm for the major axis (with 110 nm the length most frequent) and between 20 and 190 nm for the minor axis (85 nm most frequent).

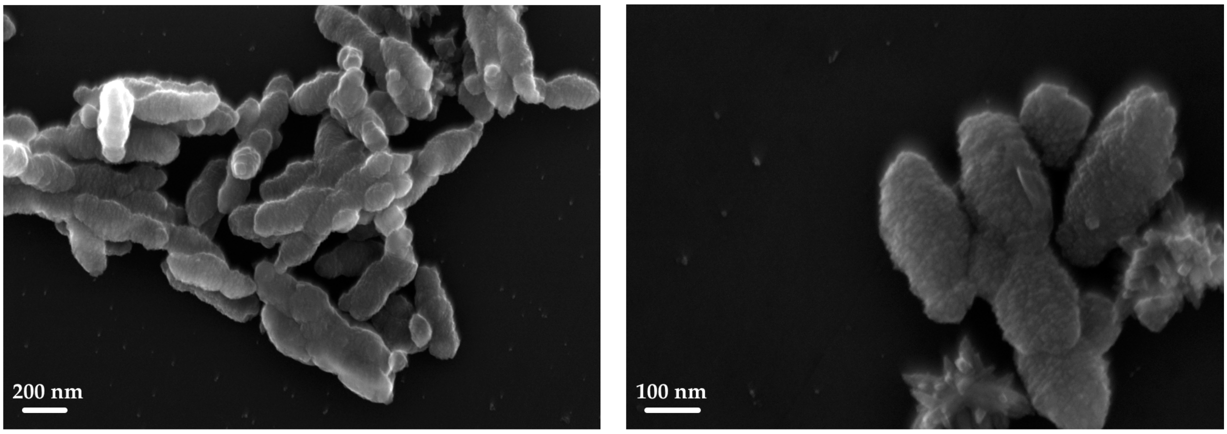

(a) **(b)**

Figure S2. SEM images of TP23.4 sample (a) and zoom (b). At *r*=125 nanostructures of elongated shape are obtained, connected to each other; a broad size distribution is obtained, with more frequent values between 315 and 450 nm (major axis) and 150-170 nm (minor axis).

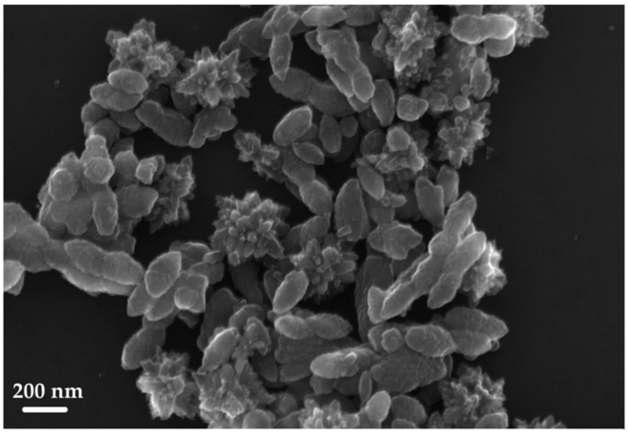

Figure S3. SEM image of TP23.5 sample. At *r* =150 the nanostructures are smaller, less elongated (the most frequent dimensions of the axes lie in intervals of 185-240 nm and 95-120 nm respectively) and less connected. The number of "star" shaped nanoparticles is higher.

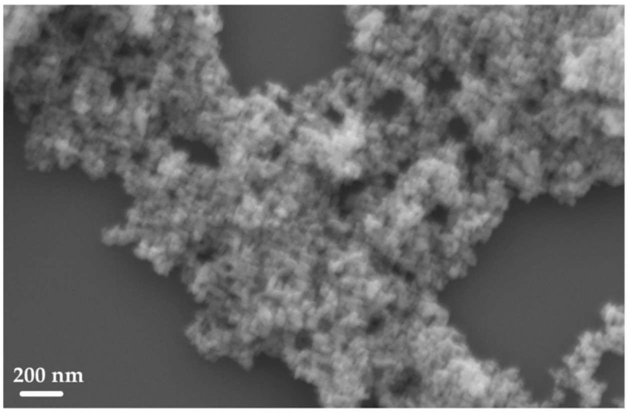

Figure S4. SEM image of TP8 sample. This sample has small nanoparticles (the most frequent between 70 and 100 nm in length and 30-60 nm in width) and aggregated in micrometric-sized structures.

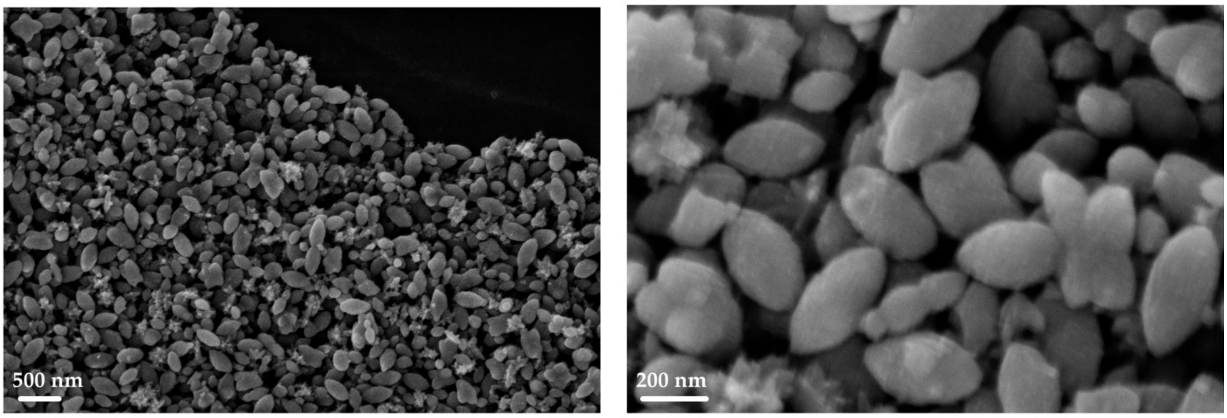

(a) **(b)**

Figure S5. SEM image of TP9 sample (a) and zoom (b). TP9 has ellipsoidal nanoparticles of wide dimensional distribution, mixed with "star" shaped structures.

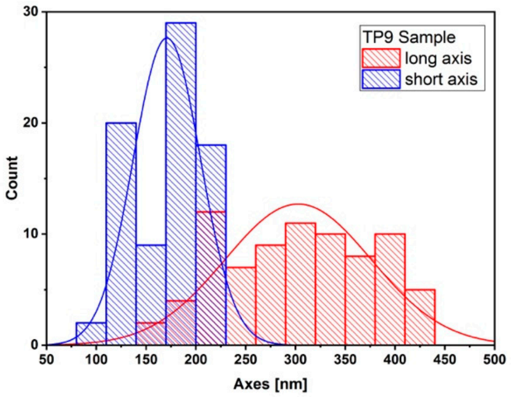

Figure S6. Histogram of TP9 sample representing the dimensional distribution of the NPs.

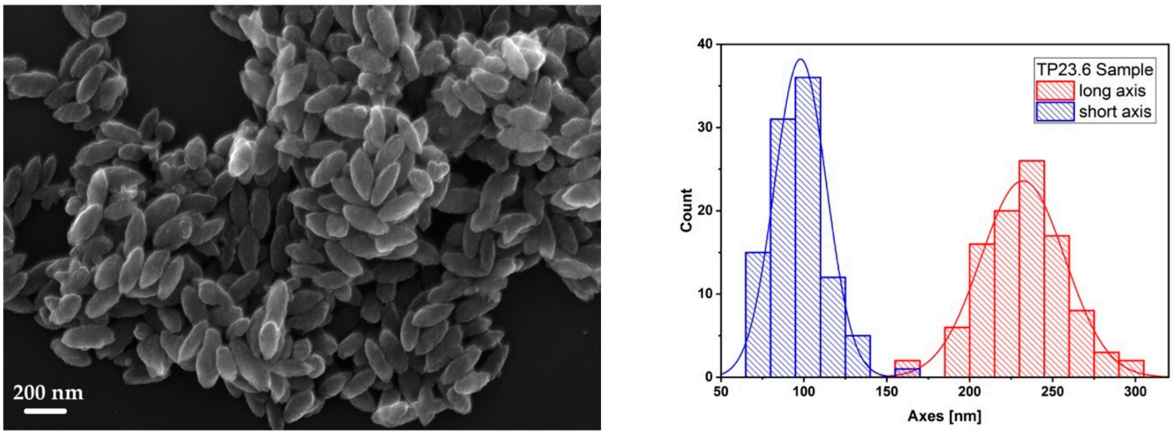

Figure S7. SEM image of TP23.6 sample (a) At r = 175 nanoparticles with a tapered "rice grain" shape and more homogeneous dimensions can be distinguished. The size distribution (b) is characterized by more frequent values of 245 nm for the major axis and 100 nm for the minor axis.

S2.2. Acid synthesis Samples

Table S5. Results with the acid synthesis using molar ratios TTIP:H_2O:HAc = 1:r:105.

Sample	$\frac{mol\ (H_2O)}{mol\ (Ti(O^iPr)_4)}$	Normalized Raman Intensity [count/mW]	Peak Position [cm^{-1}]	FWHM [cm^{-1}]	Crystallite Diameter (by Scherrer) [nm]	Notes
**TP22.8**	1.66	200197	146.61	16.86	13	
**TP22.10**	3.32	325368	146.97	16.61	13	
**TP25.1**	3.32	207889	147.24	16.57	34	
**TP27.1**	3.74	296225	147.43	17.65	13	+ Amorphous
**TP22.7**	4.15	76463	147.27	16.55	23	+ Amorphous
**TP27.2**	10.38	32664	146.49	16.02	24	+ Amorphous
**TP25.2**	16.6	27052	145.64	14.01	17	
**TP22.9**	16.6	32215	144.83	15.14	13	
**TP22.5**	41.5	31184	144.90	13.67	12	
**TP25.3**	75	32055	144.67	13.35	13	
**TP25.4**	125	31860	144.76	13.05	14	
**TP25.5**	150	26131	145.05	13.33	14	
**TP25.6**	225	25571	144.77	13.90	12	+ Amorphous
**TP25.7**	346	21954	144.73	13.17	14	+ Amorphous
**TP25.8**	708	23733	145.06	13.95	12	+ Amorphous
**TP25.9**	1431	16170	145.40	15.64	13	

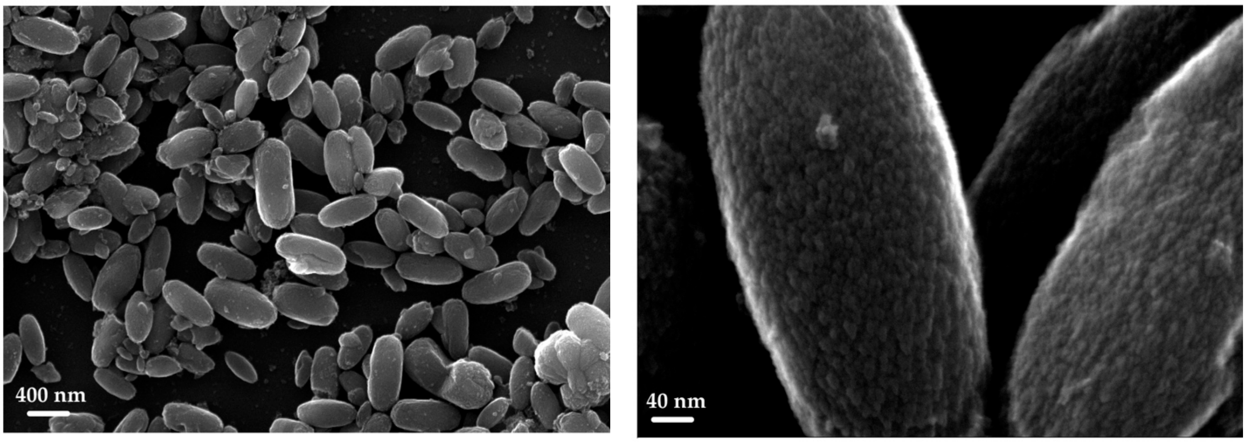

(a) **(b)**

Figure S8. SEM image of TP22.8 sample (a) zoom on a single NPs (b).

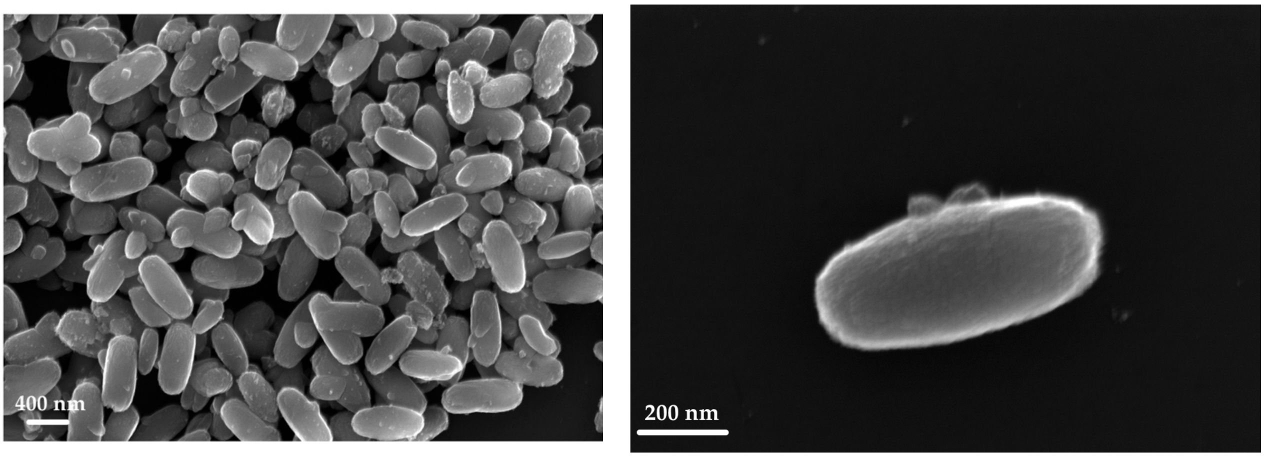

(a) **(b)**

Figure S9. SEM image of TP22.10 sample (a) and zoom on a single NPs (b).

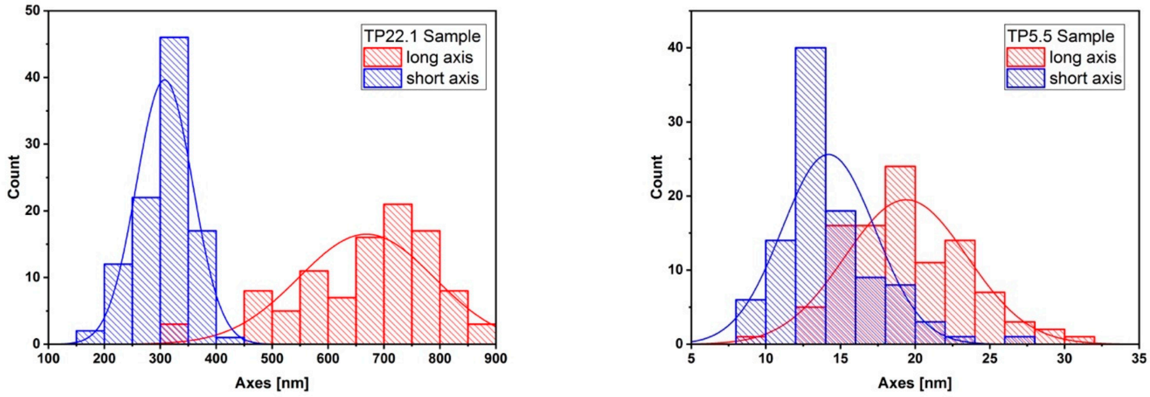

(a) **(b)**

Figure S10. Histogram of TP22.10 sample representing the dimensional distribution of the NPs.

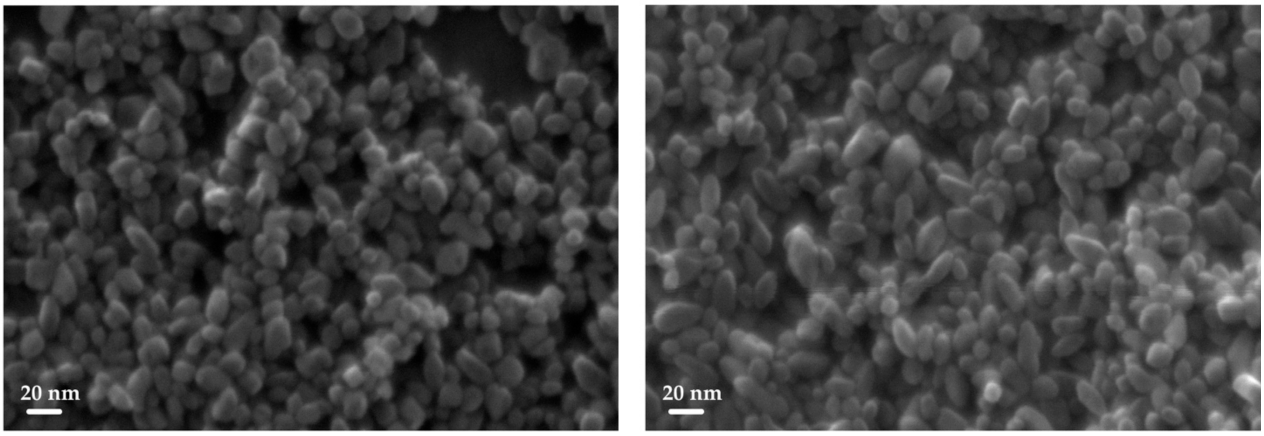

(a) **(b)**

Figure S11. SEM image of TP25.5 (a) TP25.9 (b) samples.

S2.3. Basic synthesis Samples

Table S6. Results with the basic synthesis.

Sample	$\frac{mol\ (H_2O)}{mol\ (Ti(O^iPr)_4)}$	Normalized Raman Intensity [count/mW]	Peak Position [cm^{-1}]	FWHM [cm^{-1}]	Crystallite Diameter (by Scherrer) [nm]	NOTE
**TP26.7**	16.6	61832	143.53	12.40	22	+ Brookite (traces)
**TP26.8**	41.5	55131	143.83	12.97	17	+ Brookite (traces)
**TP26.9**	75.0	80032	143.66	12.75	20	+ Brookite (traces)
**TP21.1**	128.7	26289	143.74	13.04	21	+ Brookite (traces)
**TP21.2**	164.9	30588	143.29	12.71	19	+ Brookite (traces)
**TP21.3**	225.3	38969	143.89	12.97	21	+ Brookite (traces)
**TP21.4**	345.9	36875	143.22	13.31	19	+ Brookite (traces)
**TP21.5**	707.9	33422	143.12	12.69	18	+ Brookite (traces)
**TP21.6**	1431.8	30091	143.26	13.51	18	+ Amorphous
**TP25C**	225.2	46361	143.89	12.97	23	+ Brookite (traces)
**TP25S**	225.2	32761	143.07	11.9	19	+ Brookite
**TP25T**	225.2	1083587	143.78	12.46	50	+ Rutile
**TP25T1**	225.2	257741	142.28	10.79	rut	+ Brookite + Rutile
**TP25T2**	150	903950	142.21	9.02	56	
**TP20**	21728.4	38647	142.73	10.07	23	
**TP25C**	225.2	46361	143.89	12.97	23	+ Brookite
**TP25S**	225.2	32761	143.07	11.9	19	+ Brookite

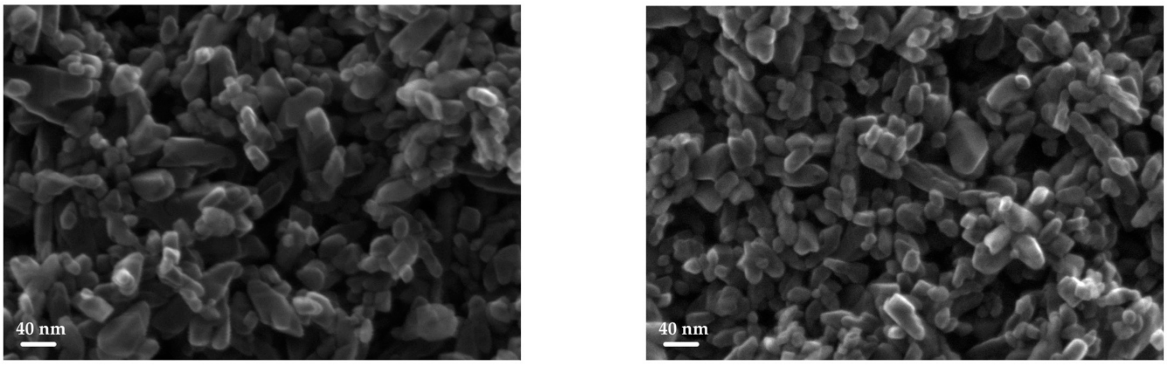

(a) **(b)**

Figure S12. SEM image of TP21.4 (a) and TP21.6 samples.

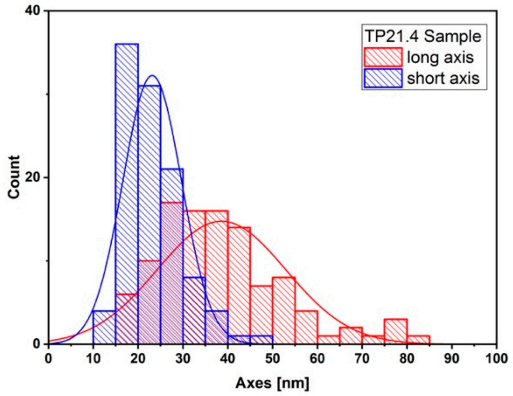

Figure S13. Histogram of TP21.4 sample representing the dimensional distribution of the NPs.

S3. Optical and morphological Characterization of Samples

Table S7. Parameters of materials tested for nanothermometry.

Sample	Normalized Intensity Eg(1) peak @ 25°C		Temperature [K]	$\frac{I_{aS}}{I_S}$		Sensitivity
**TP9**	180926	± 4591	293.2	0.492	± 0.003	0.254
			298.2	0.500	± 0.007	0.245
			303.2	0.500	± 0.006	0.240
			308.2	0.505	± 0.008	0.233
			313.2	0.511	± 0.003	0.226
			318.2	0.510	± 0.006	0.222
			323.2	0.519	± 0.003	0.214
**TP21.3**	38969	± 982	293.2	0.503	± 0.005	0.247
			298.2	0.500	± 0.002	0.243
			303.2	0.507	± 0.003	0.235
			308.2	0.511	± 0.002	0.228
			313.2	0.517	± 0.003	0.221
			318.2	0.523	± 0.003	0.214
			323.2	0.527	± 0.003	0.208
**TP22.10**	325368	± 8132	293.2	0.49	± 0.02	0.254
			298.2	0.482	± 0.004	0.254
			303.2	0.493	± 0.004	0.243
			308.2	0.496	± 0.001	0.237
			313.2	0.504	± 0.003	0.228
			318.2	0.508	± 0.001	0.222
			323.2	0.514	± 0.001	0.216
**TP25.1**	207889	± 5233	293.2	0.49	± 0.01	0.255
			298.2	0.491	± 0.003	0.249
			303.2	0.502	± 0.003	0.239
			308.2	0.503	± 0.006	0.234
			313.2	0.510	± 0.003	0.226
			318.2	0.511	± 0.001	0.221
			323.2	0.526	± 0.004	0.210
**TP26.9**	80032	± 9881	298.2	0.508	± 0.003	0.239
			303.2	0.514	± 0.003	0.231
			308.2	0.522	± 0.002	0.223
			313.2	0.526	± 0.001	0.217
			318.2	0.532	± 0.003	0.210
			323.2	0.540	± 0.002	0.203
